# First report of infections due to *Candidozyma* (formerly *Candida*) *auris* in Botswana, 2022–2024

**DOI:** 10.1128/spectrum.02042-25

**Published:** 2025-09-03

**Authors:** Teresia W. Gatonye, Tichaona Machiya, Janet John-Thubuka, Jack Mkubwa, Tlhalefo D. Ntereke, Tshiamo Zankere, Kwana Lechiile, Chimwemwe Viola Tembo, Boingotlo Gopolang, Kabelo Zwinila, Celda Tiroyakgosi, Naledi Mannathoko, Margaret Mokomane, Daniel Loeto, Miriam Mwamba, Rudzani Mashau, Ruth Mpembe, Silondiwe Nzimande, Nelesh P. Govender, David M. Goldfarb, Ebbing Lautenbach, Susan Coffin, Britt Nakstad, Jonathan Strysko, Melissa Richard-Greenblatt

**Affiliations:** 1 Botswana-University of Pennsylvania Partnership, Gaborone, Botswana; 2 Botswana Ministry of Health & Wellness 107794 , Gaborone, Botswana; 3 University of Botswana 54547, Gaborone, Botswana; 4 National Institute for Communicable Diseases 70687, Johannesburg, South Africa; 5 University of the Witwatersrand 37707 , Johannesburg, South Africa; 6 University of British Columbia, Vancouver, British Columbia, Canada; 7 University of Pennsylvania 6572, Philadelphia, Pennsylvania, USA; 8 Children’s Hospital of Philadelphia 70687, Philadelphia, Pennsylvania, USA; 9 The Hospital for Sick Children, Toronto, Ontario, Canada; 10 University of Toronto 7938, Toronto, Ontario, Canada; Mayo Foundation for Medical Education and Research, Rochester, Minnesota, USA

**Keywords:** Africa, Botswana, *Candida auris*, *Candidozyma auris*

## LETTER

Since it was first reported in Japan in 2009 (1), *Candidozyma* (formerly *Candida*) *auris* has been identified in over 60 countries across six continents (2). However, identification of *C. auris* primarily occurs in high-resource laboratory settings in which matrix-assisted laser desorption/ionization time-of-flight mass spectrometry (MALDI-TOF-MS) and molecular identification methods for *C. auris* are available. To date, only six countries in Africa have identified clinical cases of *C. auris,* likely due in part to the limited availability of necessary identification techniques and limited surveillance infrastructure (2). *C. auris* may thus be more widespread in healthcare facilities across low- and middle-income countries (LMICs) than current data suggests.

In South Africa, where diagnostic capacity, fungal pathogen identification, and *C. auris* surveillance systems are well established, over 100 hospitals have reported *C. auris* infections (3), making it the second most common cause of candidemia after *Candida parapsilosis* (1–5). To the best of our knowledge, there have been no documented cases of *C. auris* in Botswana. Given the country’s proximity to South Africa, frequent cross-border travel, and shared medical services, the possibility of undetected *C. auris* transmission has been a growing concern. Through retrospective review of stored clinical isolates, we report Botswana’s first confirmed clinical *C. auris* cases.

We reviewed 18 months of clinical data from a 530-bed hospital, including eight intensive care unit (ICU) and neonatal ICU beds, based in Gaborone, Botswana, between August 2022 and January 2024. The hospital is the only government tertiary care center in the capital. Although clinical laboratory records described a total of 222 samples with presumptive *Candida* spp. isolated in culture (1.5%; 222/14,628), only 66 (29.7%; 66/222) of these isolates were stored. All 66 isolates were sent to Wits Mycology/National Institute for Communicable Diseases in South Africa for further identification. MALDI-TOF-MS (Vitek MS, bioMérieux, France) confirmed 44 (66.7%; 44/66) isolates as *Candida* spp., of which 10 were identified as *C. auris* (22.7%; 10/44). Most cases (7/10) occurred among adults admitted to the hospital’s ICU. Comprehensive clinical data were not available for review; however, patient demographic and clinical details are outlined in Table 1.

**TABLE 1:** Demographic and clinical characteristics of patients with *C. auris* isolated from clinical specimens in a tertiary referral hospital in Botswana (August 2022 to January 2024)

Case number	Month/year	Ward	Age	Sex	Specimen source	Length of hospital stay	Length of stay prior to detection	Transferred from outside facility	Outcome
1	Aug 2022	Intensive care unit	54 years	F	Wound culture	63 days	46 days	No	Died
2	Dec 2022	Neonatal unit	21 days	M	Blood culture	57 days	19 days	No	Survived
3	Feb 2023	Intensive care unit	48 years	M	Blood culture	12 days	Unknown	Yes	Died
4	Apr 2023	Intensive care unit	66 years	M	Blood culture	53 days	19 days	No	Survived
5	Jul 2023	Intensive care unit	44 years	M	Blood culture	Unknown	122 days	Yes	Unknown
6	Jul 2023	Paediatric medical ward	36 days	M	Urine culture	Unknown	Unknown	Unknown	Unknown
7	Oct 2023	Intensive care unit	62 years	M	Blood culture	84 days	63 days	Yes	Died
8	Oct 2023	Emergency department	67 years	M	Blood culture	Unknown	Unknown	Unknown	Unknown
9	Oct 2023	Intensive care unit	54 years	F	Blood culture	5 days	11 days	Yes	Died
10	Nov 2023	Intensive care unit	30 years	M	Blood culture	99 days	94 days	No	Died

Antifungal susceptibility testing was performed on nine of the *C. auris* isolates (Table 2). All isolates were resistant to fluconazole but susceptible to echinocandins and amphotericin B using U.S. Centers for Disease Control and Prevention tentative breakpoints (6–8). Whole-genome sequencing revealed the 10 isolates to belong to the clade III lineage that was first described in South Africa (6). The number of single nucleotide polymorphisms between isolates was ≥45, which is greater than the number of previously described within-patient or within-facility differences (7, 8), suggesting multiple introduction/transmission events in our hospital rather than a single-source outbreak.

**TABLE 2:** Minimal inhibitory concentration of nine *C. auris* isolated from clinical specimens in a tertiary referral hospital in Botswana (August 2022 to January 2024)^*a*^

Case number	Minimal inhibitory concentration (μg/mL)							
	Fluconazole	Itraconazole	Voriconazole	Posaconazole	Caspofungin	Micafungin	Anidulafungin	Amphotericin B
1	128	0.12	1	0.03	0.5	0.12	0.25	0.75
2	128	0.12	0.5	0.03	0.12	0.12	0.12	0.5
3	128	0.12	0.5	0.03	0.25	0.12	0.12	0.75
4	256	0.12	0.5	0.03	0.25	0.12	0.12	0.75
5	128	0.12	0.5	0.03	0.25	0.12	0.25	0.75
7	256	0.5	2	0.5	0.25	0.06	0.25	1
8	256	0.12	2	0.06	0.5	0.25	0.12	1
9	256	0.12	1	0.06	0.5	0.25	0.25	0.75
10	128	0.12	1	0.06	0.12	0.06	0.12	0.75

^*a*^ Antifungal susceptibility testing was performed using a commercial broth microdilution assay (Sensititre, Thermo Fisher, Waltham, MA, USA) for all agents with the exception of amphotericin B, which was performed using an Etest (bioMérieux, Marcy-l'Étoile, France). Minimal inhibitory concentration interpretations were based on the U.S. Centres for Disease Control and Prevention tenative breakpoints (5): fluconazole (≥32 μg/mL), caspofungin (≥2 μg/mL), micafungin (≥4 μg/mL), anidulafungin (≥4 μg/mL), and amphotericin B (≥2 μg/mL). No breakpoints are available for other second-generation triazoles; however, fluconazole susceptibility is considered a surrogate.

This letter documents the first confirmed clinical cases of *C. auris* in Botswana, expanding the known geographic distribution of this organism in southern Africa. These findings highlight the urgent need for enhanced fungal diagnostic capacity and robust surveillance systems in LMICs, which remain vulnerable to undetected transmission and outbreaks. Strengthening laboratory infrastructure, fostering regional collaboration, and integrating *C. auris* detection into existing antimicrobial resistance frameworks are imperative to mitigate its impact.
